# Trisomy 13 with retroiliac ureter diagnosed by assessment for recurrent febrile urinary tract infections

**DOI:** 10.1002/iju5.12692

**Published:** 2024-01-23

**Authors:** Yosuke Morizawa, Mitsuru Tomizawa, Takuto Shimizu, Kenta Onishi, Shunta Hori, Daisuke Gotoh, Yasushi Nakai, Makito Miyake, Kazumasa Torimoto, Kiyohide Fujimoto

**Affiliations:** ^1^ Department of Urology Nara Medical University Kashihara Nara Japan

**Keywords:** hydronephrosis, mid‐ureteral stricture, retroiliac ureter, trisomy 13, ureteroureterostomy

## Abstract

**Introduction:**

Patients with trisomy 13 have multiple malformations, including urological anomalies, and severe cognitive and psychomotor disabilities. We conducted a ureteroureterostomy for a mid‐ureteral stricture due to a retroiliac ureter in a patient with trisomy 13.

**Case presentation:**

A 6‐month‐old girl with trisomy 13 developed a urinary tract infection. Computed tomography for assessing recurrent urinary tract infection revealed a left mid‐ureteral stricture due to the retroiliac ureter. At the age of 2, a ureteroureterostomy was performed. Two years after surgery, the urinary tract infection did not recur.

**Conclusion:**

Ureteroureterostomy is a safe procedure for children with trisomy 13 and multiple comorbidities. Surgical treatment should be considered for patients with trisomy 13 when agreed upon by the family and comorbidities are well‐controlled.

**Abbreviations & Acronyms**
CT: computed tomography
UTI: urinary tract infection
VUR: vesicoureteral reflux

**Keynote message**

We reported the first case of trisomy 13 associated with the retroiliac ureter. The ureteroureterostomy we performed in this case on the patient was safe and effective. Aggressive treatment such as urinary reconstruction surgery should be considered for patients with trisomy 13 provided the family agrees and the comorbidities are controlled.

## Introduction

Trisomy 13 is an autosomal aneuploidy of chromosome 13, first described by Patau *et al*.^1^ Estimating the actual prevalence of T trisomy 13 is difficult because of high rates of spontaneous abortions, medical abortions, and intrauterine fetal deaths. Infants with trisomy 13 have a median survival of 7–10 days.^2^ Favorable prognostic factors for trisomy 13 include female sex, African American ancestry, mosaicism, and the absence of congenital heart disease.^3^ Because of the limited survival time of trisomy 13 patients, long‐term complications in surviving children have not been extensively studied. Cardiopulmonary arrest, congenital heart disease, and pneumonia are the most common causes of death in patients with trisomy 13.^4^ Recent studies estimate the 1‐year survival rate of individuals with trisomy 13 at approximately 20%, and those who survive their first year of life have a 65% probability of surviving until 10 years of age.^5^ This case with trisomy 13 suggests the validity of aggressive treatment for mid‐ureteral stricture owing to the retroiliac ureter.

## Case presentation

**Fig. 1:**
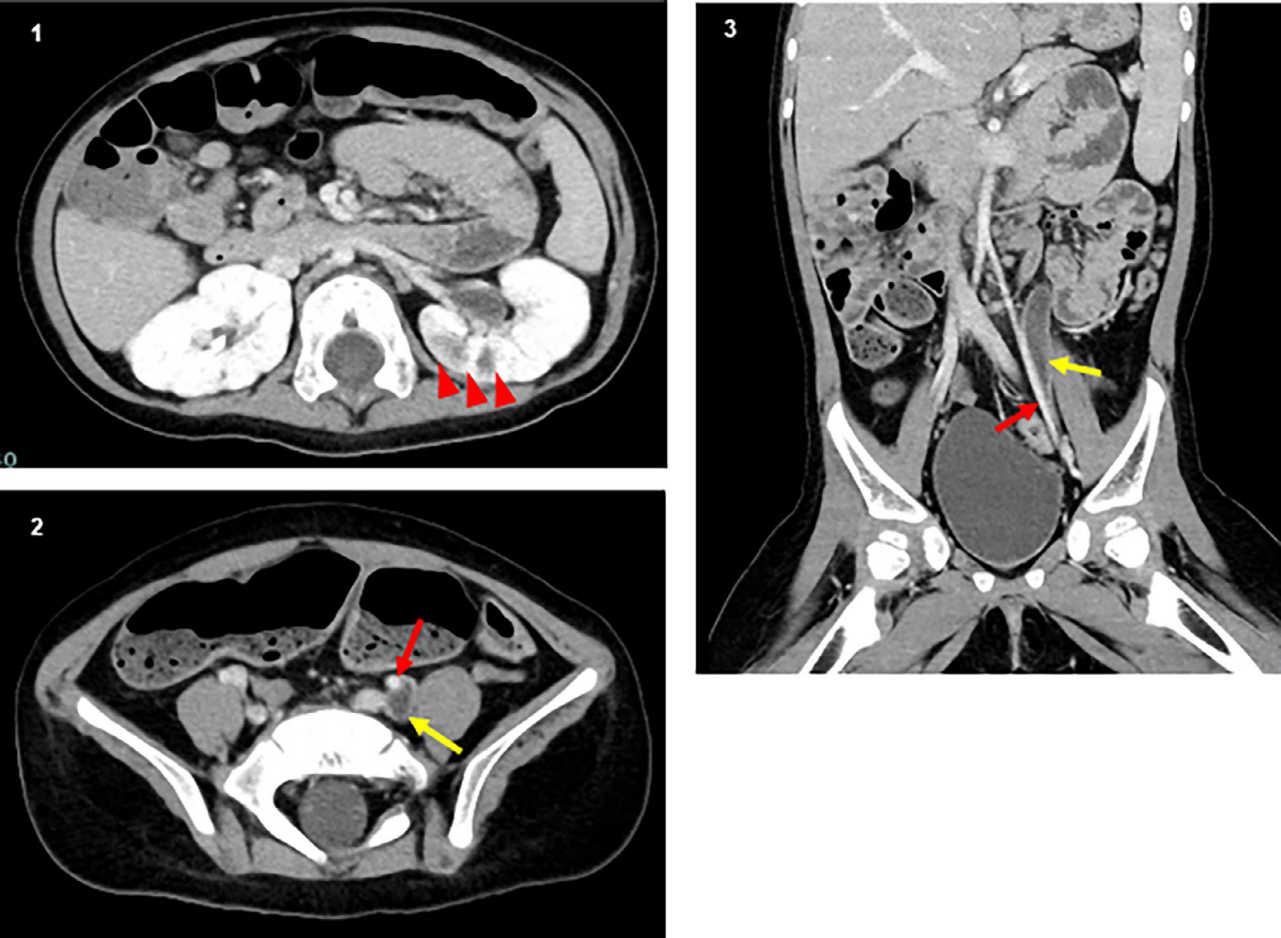
(1) Contrast‐enhanced CT showing acute focal bacterial nephritis in the left kidney (red triangles). (2 and 3) Contrast‐enhanced CT showing the retroiliac ureter. Left ureter courses posterior to the left iliac artery. Yellow arrow: hydroureter; red arrow: left iliac artery.

**Fig. 2:**
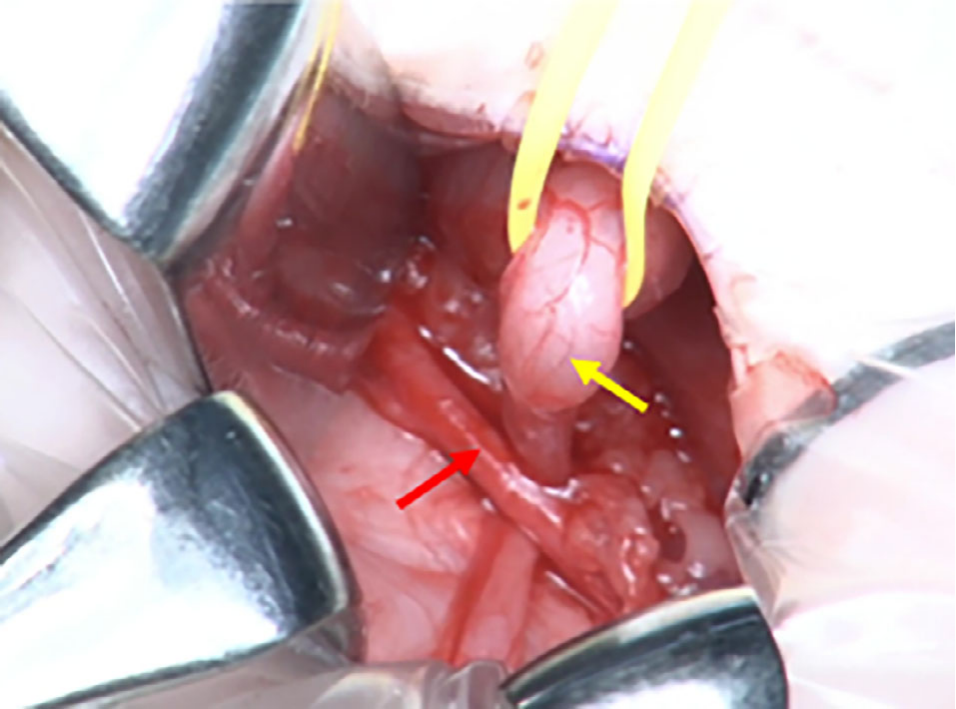
Intraoperative photograph in ureteroureterostomy. Hydroureter courses posterior to the left iliac artery. Yellow arrow: hydroureter, Red arrow: left iliac artery.

A 6‐month‐old girl with trisomy 13 developed a UTI. The patient presented with microphthalmia, tracheomalacia, apnea, central nervous system abnormalities, laryngomalacia, epilepsy with myoclonic seizures, and mild bilateral congenital hydronephrosis. Intermittent catheterization was initiated at 8 months of age after the second UTI. She developed recurrent UTIs at 14 and 16 months of age (2 years of age) following the initiation of intermittent catheterization. Contrast‐enhanced CT revealed acute focal bacterial nephritis in the left kidney and a mid‐ureteral stricture owing to the retroiliac ureter (Fig. 1‐1, 2, 3). Urodynamic studies demonstrated no detrusor overactivity and bladder compliance was 13 mL/H_2_O. Bladder capacity was 68 mL and no VUR was observed. We performed ureteroureterostomy after providing the parents with a full explanation and obtaining informed consent. Open ureteroureterostomy was performed ventral to the iliac artery after dorsal incision of the obstructed ureter through a retroperitoneal approach via a 3.5 cm Pfannenstiel skin incision (Fig. 2). The patient was discharged from the hospital 4 days after surgery without any perioperative complications. Two years after surgery, the UTI did not recur, and intermittent catheterization was discontinued.

## Discussion

We report the case of a girl with trisomy 13, recurrent UTI, and controlled respiratory and gastrointestinal complications. Urinary tract management for clean intermittent catheterization was initiated by the patient's family without invasive urological examination. In the real world, invasive procedures may be postponed, and makeshift first‐aid treatment is initiated for children with severe disabilities and multiple comorbidities, such as trisomy 13. A subsequent video‐urodynamic study showed no obvious abnormalities, while contrast‐enhanced CT revealed a mid‐ureteral stricture because of the retroiliac ureter. CT can also detect acute pyelonephritis and is faster than DMSA scanning.^6^ Once lower urinary tract dysfunction and VUR have been ruled out, detailed imaging studies, such as CT and MRI, should be performed to work up urinary tract malformations and lower urinary tract dysfunction.

Hwang reported in an autopsy of a sequence of 12 080 children that 72 children had ureteral strictures and only three children (4%) had mid‐ureteral stricture.^7^ Meng reported in a study that the incidence of mid‐ureteral stricture was in 26 patients (1.6%) in 1625 children who underwent surgical treatment for obstructive hydroureter over the past 13 years.^8^ Congenital mid‐ureteral stricture in children can be accompanied by urological abnormalities such as agenesis or atrophy of the contralateral kidney, VUR, ureteropelvic junction obstruction, crossed renal ectopia, solitary kidney, and contralateral cecum ureter.^9^, ^10^, ^11^ The retroiliac ureter, first described in 1960,^12^ is often asymptomatic or associated with other genitourinary malformations, secondary to vascular malformations similar to the retrocaval ureter.^13^, ^14^ Surgical treatment generally involves transecting the ureters on the dorsal side of the intersecting iliac arteries and performing ureteroureteral anastomosis on the ventral side.^15^ Congenital mid‐ureteral stricture is rare, and renal ultrasound alone is not reliable for demonstrating the site of obstruction.^7^ Retrograde pyelography performed during the surgical procedure is crucial for identifying the narrowing site in the affected ureteral segment. Similarly, contrast‐enhanced CT is sufficient for diagnosing mid‐ureteral stricture with associated vascular abnormalities.^16^

Here, we present the first reported case of trisomy 13 associated with the retroiliac ureter. The patient was diagnosed with a mid‐ureteral stricture due to the retroiliac artery as the cause of her UTI based on video‐urodynamics and contrast‐enhanced CT. Excision of the ureteral stricture and primary ureteroureterostomy are considered the gold standard management for mid‐ureteral stricture with established safety.^7^ Although laparoscopic surgery is a minimally invasive surgical option, we selected open ureteroureterostomy owing to concerns about changes in pulmonary function due to the intraperitoneal approach^17^ and prolongation of operative time. Pulmonary changes are of concern in infants undergoing laparoscopic surgical procedures. The transretroperitoneal open surgery we performed was a safe, effective approach for urinary tract reconstructive surgery. Brittany H. Cook reported that patients with trisomy 13 undergoing surgery exhibit frequent morbidity and an elevated, although not prohibitive, risk of death compared to patients with similar comorbidities; however.^18^ There are few reports of urinary reconstruction surgery for trisomy 13. Trisomy 13 is associated with a short long‐term prognosis; however, aggressive treatment of each complication has been reported to improve prognosis.^19^ The ureteroureterostomy we performed in this case on the patient was safe and effective. Surgeons should thoroughly counsel parents of children with trisomy 13 about these risks before deciding to undergo surgery.^18^ Aggressive treatment should be considered for patients with trisomy 13 provided the family agrees and the comorbidities are controlled. In this case, it was unclear whether hydronephrosis due to the retroiliac ureter was truly the cause of the UTI. However, since no UTI developed postoperatively, it was thought the hydronephrosis had been caused by the retroiliac ureter.

## Author contributions

Yosuke Morizawa: Conceptualization; data curation; investigation; project administration; writing – original draft; writing – review and editing. Mitsuru Tomizawa: Data curation. Takuto Shimizu: Data curation. Kenta Onishi: Data curation. Shunta Hori: Data curation. Daisuke Gotoh: Supervision. Yasushi Nakai: Supervision. Makito Miyake: Supervision. Kazumasa Torimoto: Supervision; writing – original draft; writing – review and editing. Kiyohide Fujimoto: Supervision.

## Conflict of interest

The authors declare no conflicts of interest.

## Approval of the research protocol by an Institutional Reviewer Board

This study complied with the 1964 Declaration of Helsinki and its later amendments and comparable ethical standards.

## Informed consent

Not applicable.

## Registry and the Registration No. of the study/trial

Not applicable.

## Animal studies

Not applicable.

## Funding information

This study did not receive any specific grants from funding agencies in the public, commercial, or non‐profit sectors.
